# Interpersonal and communication skills development in nursing preceptorship education and training programmes: a scoping review protocol

**DOI:** 10.12688/hrbopenres.13201.2

**Published:** 2021-03-11

**Authors:** Philip Hardie, Andrew Darley, Catherine Redmond, Attracta Lafferty, Suzi Jarvis

**Affiliations:** 1 UCD School of Nursing, Midwifery and Health Systems, University College Dublin, Belfield, Dublin 4, Ireland; 2 UCD School of Medicine, University College Dublin, Belfield, Dublin 4, Ireland; 3 UCD Innovation Academy, University College Dublin, Belfield, Dublin 4, Ireland

**Keywords:** Interpersonal and Communication Skills, Soft Skills, Nursing Preceptorship, Preceptorship Education and Training, Pedagogy

## Abstract

The preceptorship model is an education-focused model for teaching and learning within a clinical environment in nursing. It formulates a professional educational relationship between a staff nurse (preceptor) and student nurse and is based on the provision of providing patient care. Preceptorship is widely acknowledged in the literature as a positive pedagogical approach in clinical nursing education in terms of knowledge and skill acquisition, confidence, and professional socialisation of undergraduate nursing students. However, the literature also widely reports negative interpersonal experiences within this professional educational relationship resulting in negative educational experiences and in some cases, negative patient experiences. Therefore, the authors set out to examine what teaching strategies are being implemented by nurse educators to encourage the development of interpersonal and communication skills in facilitating positive interpersonal relationships between the preceptor, nursing student and patient. This paper outlines the protocol for an exploratory scoping review that aims to systematically and comprehensively map out the available published and unpublished literature on the teaching strategies to develop interpersonal and communication skills in preceptorship education and training programmes. To conduct a systematic and comprehensive scoping review, the review will be guided by the Joanna Briggs Institute and Arksey & O’ Malley (2005) six-stage iterative framework, as well as PRISMA-ScR framework guidelines, to ensure the quality of the methodological and reporting approaches to the review. It is anticipated that the results of the scoping review will inform nurse educators on the current educational practices for developing interpersonal and communication skills in preceptorship education and training programmes and identify any educational practices that are worthy of further consideration for future research.

## Introduction

The preceptorship model is a teaching and learning strategy frequently employed internationally to educate undergraduate and graduate nursing students in the clinical environment. Some debate exists regarding the definition and function of preceptorship, for example Billay & Myrick’s (2008, pg. 259) defines preceptorship as:

“ *an approach to the teaching and learning process within the context of the practice setting which allows students to develop self-confidence while increasing their competence as they become socialised into the profession of nursing*”.

Carlson (2013, pg.457) further defines preceptorship “ *as a trusting relationship between preceptor and student.... where the preceptor strives to create a safe and meaningful interactive relationship with the student...... thus supporting the student’s ability to implement generalized theoretical knowledge into patient-centered problems*”. Another key function of a preceptorship not captured in the previous definitions is the preceptor’s role in the assessment of students’ competencies. Vae *et al.*, (2018, pg.13) states “ *the students' learning process is dependent on high-quality assessment processes and feedback from preceptors permitting students to critically reflect on their practice and learn for that experience*”.

Acknowledging that up to 50% of undergraduate nursing curriculums take place in the clinical environment, the preceptorship model plays a pivotal role in the education of student nurses ( McSharry & Lathlean, 2017; NMBI, 2016a; NMC, 2018). The preceptorship relationship is a purposeful short-term professional partnership in the practice setting between the preceptor (staff nurse) and a nursing student (preceptee) and is based on the provision of providing patient care. Hence, within the preceptorship model, there are three members: the preceptor, the student nurse, and the patient, forming a triadic professional relationship. This relationship provides opportunities for nursing students’ socialisation into nursing practice and helps to integrate theory into practice, under the guidance of the preceptor ( McSharry & Lathlean, 2017; Muir *et al*., 2013; Ward & McComb, 2017). The Nursing and Midwifery Board of Ireland ( NMBI, 2016b) states that effective interpersonal relationships are not only essential for the foundation of effective patient care but also a successful teaching and learning environment. Therefore, the quality and support within this relationship contribute significantly to nursing student’s socialisation into the nursing profession and their learning ( Ward & McComb, 2017) whilst also ensuring quality patient care and their satisfaction in their nursing care ( Suikkala *et al*., 2020a). Thus, the formation of a therapeutic interpersonal relationship between the preceptor/nursing student and patient is essential.

Kornhaber *et al*. (2016 pg. 537) define a therapeutic interpersonal relationship between the nurse and patient as:

“ *a relationship which is perceived by patients to encompass caring, and supportive non-judgmental behaviour, embedded in a safe environment during an often-stressful period*”.

Typically, a positive therapeutic relationship portrays characteristics of good interpersonal competence with meaningful dialogue that displays warmth, friendliness, genuine interest, respect, and empathy, while at the same time responding to patients’ emotions and having a desire to provide support and care ( Dinç & Gastmans, 2013; Kornhaber *et al*., 2016; Prip *et al*., 2018). It is therefore essential that preceptors are proficient in these skills, which will enable them to provide patient-centered care, while also utilising teaching techniques such as role modelling, coaching and contextual questioning to facilitate the student’s learning ( McSharry, 2013). For this reason, there needs to be a strong educational and trustful professional relationship between the preceptor and student nurse, so that positive therapeutic interpersonal relationships can then be developed with patients. In light of the increasing complexity of healthcare delivery, the importance of effective interpersonal relationships between the preceptor, nursing students and patients has grown exponentially. The literature emphasises the benefits of effective interpersonal and communication skills but also highlights the consequences of negative interpersonal interactions. Effective interpersonal relations have been shown to play a pivotal role in building trusting relationships and creating a caring and welcoming environment in nursing ( Arnold & Boggs, 2020). This thereby improves communication between the preceptor, nursing student and patient, leading to person-centred care, patient satisfaction, patient empowerment and decreases adverse events among patients ( Suikkala *et al*., 2020a; Suikkala *et al*., 2020b). Effective interpersonal relations also facilitate the development of knowledge, skills acquisition, and theory-practice integrations for nursing students ( Irwin *et al*., 2018; Ke *et al*., 2017; Omer & Moola, 2019; Ward & McComb, 2017; Washington, 2013).

However, the literature to date also widely documents negative interpersonal relationships within this professional educational relationship and the clinical environment. Reports from both preceptors and nursing students exist regarding workplace incivilities such as rudeness, humiliation, anger, generational clashes, and inappropriate criticism from experienced preceptors ( Dyess & Sherman, 2009; Gardiner & Sheen 2016; Omer & Moola, 2019). These challenging experiences consequently result in a negative learning experience for the student and, in some cases, interpersonal conflict occurs due to a breakdown in the preceptorship relationship ( Hugo & Botma, 2019; McCloughen & Foster, 2018). Furthermore, it has been reported that displaying poor interpersonal skills, where there is a lack of emotional intelligence demonstrated by preceptors and student nurses, can result in negative feelings amongst patients, leading to a lack of trust and a possible breakdown in the relationship ( Holst *et al*., 2017; Mukumbang & Adejumo, 2014). As educators, this is concerning to the authors, as fragmented relationships can have not only a negative impact on the student nurses’ learning experience but also on patient care ( Cho *et al*., 2017; Suikkala *et al*., 2018; Suikkala & Leino-Kilip, 2005).

As with any human skill, interpersonal and communication skills, also referred to as “soft skills” can be improved through conscious effort ( Moss, 2020). McConnell (2004, pg. 178) describes soft skills as “ *those essential skills involved in dealing with and relating to other people, largely on a one-to-one basis*”. These include the ability to engage with others at a personal and professional level, and display levels of empathy towards the situation that others may be experiencing ( Grant & Goodman, 2019). This process stimulates feelings of support, comfort, and recognition in individuals ( Wright, 2007). Enhancement of interpersonal skills concerns several key components, including the individuals’ emotional intelligence, learning to recognise the uniqueness of everyone, empathising with the individual, learning to listen, effective communication, empowering others and building trust ( Grant & Goodman, 2019).

Teaching effective interpersonal and communication skills requires providing the relevant knowledge, as well as guiding and coaching learning to develop and enhance these skills. It takes time and experience to build effective interpersonal and communication skills, beginning with foundational skills, for example, knowing when to use open-ended and closed-ended questions. More advanced listening skills paired with sensitivity and empathy generate highly effective interpersonal relationships ( Pavord & Donnelly, 2015). Investing in developing preceptors’ interpersonal and communication skills is essential in maintaining good interpersonal relationships, an effective teaching environment and exemplary patient care. Preceptors not only have the responsibility of role modelling effective soft skills but also evaluating student nurses’ competencies in these skills as part of their clinical assessment document ( NMBI, 2016a). Therefore, the inclusion of such skills is paramount in preceptorship education and training programmes. Preceptors bring their own distinctive set of communication skills, cultural influences, learning styles and life experiences that directly affect their ability to engage in effective interpersonal relationships ( Gardiner & Sheen, 2016). Nurse educators must build on preceptors’ strengths and experiences to enhance their interpersonal skills. A preceptor short of adequate interpersonal and communication skills may be able to facilitate positive interpersonal relationships with the nursing students and patients ( Martínez-Linares *et al*., 2019). Interpersonal and communication skills are practical skills. Therefore, nursing educators need to adapt teaching strategies that involve activities which allow opportunities for active participation to develop such skills, e.g., experiential learning opportunities ( Reid-Searl *et al*., 2017). Pedagogical approaches observed in the literature include simulation practices such as the use of puppets ( Reid-Searl *et al*., 2017), standardised patients ( Lin *et al*., 2013; Maclean *et al*., 2017), real patients ( Perry *et al*., 2013) and roleplay ( Jackson & Back, 2011; Pearson & McLafferty, 2011). Other methods observed included clinical placement ( Purdie *et al*., 2008), group discussions ( Waugh *et al*., 2014), online discussion ( Deering & Eichelberger, 2002), and audiotapes ( Sloan, 2003).

The existing evidence outlined provides an overview of the importance of interpersonal and communication skills, particularly in the context of a nursing preceptorship relationship. This literature highlights the need for active development of these skills in preceptorship education and training programmes. However, an initial inspection of the literature demonstrates that the focus of interpersonal and communication skills development centres around nurse-patient relationships and is predominantly completed as part of an undergraduate nursing programme. Given the importance of effective interpersonal and communication skills for preceptors in not only facilitating and guiding such skills among nursing students and the patients, the authors feel it is therefore worthwhile to systematically examine the literature to identify what teaching strategies are being implemented to develop interpersonal and communications skills among trainee preceptors (qualified nurses). It is also important to determine if trainee preceptors are being afforded the opportunity to specifically develop interpersonal and communication skills required to facilitate and guide the triadic preceptorship relationship between the nurse, student nurse and patient.

A scoping review protocol will outline the approach that will be adopted to determine the available literature on the pedagogical approaches to developing interpersonal and communication skills among nursing preceptors as part of their preceptorship education and training programme.

## Protocol

An exploratory scoping review approach will be employed to establish the nature and extent of knowledge relating to pedagogical approaches in preceptorship education and training programmes for the development of interpersonal and communications skills among trainee preceptors. Scoping reviews are used to map the concepts underpinning a research area and the primary sources and types of evidence available ( Arksey & O’Malley, 2005). Scoping reviews present a broad overview of the evidence concerning a topic and are beneficial when investigating areas that are emerging to clarify key concepts, definitions and identify gaps ( Lockwood *et al*., 2019; Page & Moher, 2017). Scoping reviews are also implemented to examine the breadth of the literature, as well as the conduct of research on a specific topic to inform the design of future research studies ( Lockwood *et al*., 2019).

To the best of the authors’ knowledge, there are currently no scoping reviews examining the educational practices of nurse educators in developing interpersonal and communication skills among trainee preceptors. Therefore, the findings of this exploratory review will contribute to existing literature regarding current pedagogy for interpersonal and communications skills development in preceptorship education and training programmes. The findings of this review may benefit the wider society, considering that interpersonal relationships of a preceptorship not only play an essential role in providing effective patient care, but also in facilitating nursing education in the clinical environment. It will also contribute a theoretical and empirical basis for the future development of pedagogical approaches that aim to enhance interpersonal and communication skills in preceptorship education and training programmes. The scoping review protocol introduced by Arksey & O’Malley (2005) that encompasses a six-stage iterative framework, as well as Peters *et al*. (2020) updated approach to conducting scoping reviews, will guide this review protocol and subsequent scoping review. 

### Aim and objectives

The overall aim of the scoping review is to identify, explore and map the literature regarding the development of interpersonal and communication skills for preceptors as part of their preceptorship education and training programme.

This will be achieved by addressing the following objectives:

1. Determine the extent and nature of existing literature on the development of interpersonal and communication skills in preceptorship education and training programmes, so that the literature can be examined and mapped out, to identify any gaps.
2. Examine current educational practices for the development of interpersonal and communication skills in preceptorship education and training programmes, to identify any educational practices that are worthy of further consideration for future research.

### Stage 1: Identification of the scoping review research question

Peters *et al*. (2020) state that a clear scoping review question should incorporate elements of the PCC mnemonic (population, concept, and context). In this instance, qualified nursing staff (trainee preceptors/preceptors) are the relevant population, the concept is educational practices for interpersonal and communication skills development for supporting newly qualified nurses taking part in preceptorship programmes as well as undergraduate nursing students, and the context is preceptorship education and training programmes in acute/residential clinical settings. The research question that will therefore guide this scoping review is:

“ *What is known about the development of interpersonal and communication skills amongst trainee nursing preceptors in preceptorship education and training programmes?*”

### Stage 2: Identifying relevant studies

The Preferred Reporting Items for Systematic reviews and Meta-Analysis extension for Scoping Reviews (PRISMA-ScR) Checklist framework guidelines introduced by Tricco *et al*. (2018) will also guide the systematic scoping review. These guidelines are recommended to enhance transparency and quality of the completed scoping review ( McGowan *et al*., 2020), and will help the researchers and the readers develop a greater understanding of the evidence. The research team will undertake a comprehensive search of the literature within the following databases:

- CINAHL Nursing and Allied Health (CINAHL Plus)
- SCOPUS (Elsevier Publications)
- Academic Search Complete (EBSCOhost)
- APA PsycINFO (American Psychological Association)
- Education (ERIC)

Joanna Briggs Institute (JBI) three-step process for applying a search strategy will be implemented ( Peters *et al*., 2020). Firstly, an initial search was deployed on CINAHL Plus, the identification of search terms was conceptually based on an oriented search to identify key text words used to address the major concepts which include population (preceptors), concept (interpersonal and communication skills development), and context (nursing preceptorship education and training programme). Synonyms for each of the concepts will also be included. Each search strategy will be adapted to the functionality of each database using specific Boolean operators, truncation markers, and MeSH headings where necessary to broaden the search and capture all literature that may use such terms. McGowan *et al*. (2020) state the input of a research librarian is invaluable when carrying out a scoping review; the authors worked with an expert university librarian (D.S.) in designing and refining the search strategy. Table 1 outlines the keywords for each search string. 

**Table 1.** Keywords for each search string.

**1. Population (P):** Nurse (or) Preceptor (or) Mentor (or) Supervisor (or) Practice Supervisor (or) Clinical supervisor (or) Practice assessor (or) Nursing tutor (or) Nursing instructor (or) Nursing educator
**2. Concept (C)**: Interpersonal skills (or) Soft skills (or) People skills (or) Social skills (or) Communication skills (or) Interpersonal communication (or) Verbal communication (or) Non-verbal communication (or) Active listening (or) Emotional intelligence (or) Patience (or) Negotiation (or) Conflict resolution (or) Problem-solving (or) Decision-making (or) Assertiveness (or) Teamwork (or) Humour (or) Empathy (or) Feedback
**3. Context (Co)**: Education (or) Training (or) Workshops (or)Training program (or) Educational activity (or) Training technique (or) Teaching method (or) Teaching activity (or) Pedagogy (or) Nursing education
Results from strings 1, 2, 3 will be combined to reveal a full list of articles to be initially screened by title and abstract

As per the second stage of the JBI search strategy protocol, the same keywords from Table 1 will be searched in the remaining aforementioned databases. During this stage, the research team will review and ‘hand search’ the reference list to identify any additional relevant studies. Given that this is an exploratory scoping review, the authors are interested in identifying all literature including RCTs, exploratory studies and discussion papers. Therefore a “web search” of the grey literature will also be conducted using “OpenGrey” and “Google Scholar”. Specific educational policy publications by regulatory and professional bodies for preceptorship education and training programmes will also be searched to examine the focus of interpersonal and communication skills required for a preceptorship role. Table 2 outlines the search terms for grey literature and regulatory and professional bodies for preceptorship education and training programmes.

**Table 2.** Grey literature search strategy.

Source and Link	Search terms
Open Grey http://www.opengrey.eu/ In subject: Humanities, psychology, and social sciences	Preceptorship interpersonal and communication skills Education and Training (First 10 pages)
Google Scholar https://scholar.google.com/	Preceptorship interpersonal and communication skills Education and Training (First 10 pages)
Nursing and Midwifery Board of Ireland (NMBI) https://www.nmbi.ie/Standards-Guidance	Preceptorship Interpersonal relationships Interpersonal and communication skills
Nursing and Midwifery Council (England) https://www.nmc.org.uk/	Preceptorship/Mentorship/Practise Supervisors Interpersonal relationships Interpersonal and communication skills
Health Education England https://www.hee.nhs.uk/our-work/preceptorships	Preceptorship/Mentorship/Practise Supervisors Interpersonal relationships Interpersonal and communication skills
Nursing and Midwifery Board of Australia https://www.nursingmidwiferyboard.gov.au/	Preceptorship Interpersonal relationships Interpersonal and communication skills
Canadian Nurses Association https://www.cna-aiic.ca/en	Preceptorship Interpersonal relationships Interpersonal and communication skills

### Stage 3: Study selection

Each search conducted will be systematically documented (date, search terms, results per string) and saved by two independent authors (PH, AD), with the findings of the searches compared and then imported into Mendeley (1.19.6 / 2020), a bibliographic reference manager, where any duplicates of literature will be removed before the initial screening of title and extract is divided out and screened by all of the authors. Covidence screening and data extraction software tool ( www.covidence.org) will be utilised by the authors for screening. Each article will be required to be approved by two independent screeners before either being included or excluded in the review. A pilot testing of articles (n=50) using Covidence software package and inclusion and exclusion criteria will be undertaken by the authors to ensure consistency of the methodology adopted in the selection process ( Peters *et al*., 2020). Full text screening will then be carried out on all articles that meet the inclusion criteria during the initial screening round by two independent authors (PH, CR). For any articles in which a disagreement may arise a third independent author (AL) known as the “tie-breaker” will further review the article against the inclusion criteria to settle the difference of opinion. The number of articles identified, screened, assessed for eligibility, and included in the review will be captured using the Covidence software package. A PRISMA flow diagram will be created to ensure transparency of reporting, decisions for the exclusion of studies permitting replication and comparison of any further studies.

The inclusion and exclusion criteria, highlighted in Table 3, will be developed through an iterative process based on the PCC elements of the review question, plus a specification of the types of studies that have addressed the scoping review question and discussions amongst the authors ( McKenzie *et al*., 2020). The primary author will record any changes. All authors will utilise and adhere to its criteria during the screening process to ensure consistency. 

**Table 3.** Inclusion and exclusion criteria for scoping review.

Inclusion	Rationale
Articles written in the English Language	Searches will be limited to English language due to increased resource challenges concerning costs, time, and expertise in non- English languages.
Publications between 2000 and 2020	The search will be conducted for literature published within the last twenty years.
Peer-reviewed empirical studies with either qualitative or quantitative data, mixed methods, reviews, book chapters and grey literature with a principal focus on the development of interpersonal and communication skills in preceptorship education and training programmes.	The focus of the review is to examine the development of interpersonal and communication skills in preceptorship education and training programmes; Peer-reviewed empirical studies will provide reliable and a high standard of evidence. Grey literature will capture unpublished works and local evaluation of preceptorship education and training practices.
Exclusion	Rationale
Non-English Language Studies	The English language is the primary language of the research team. Therefore, all non-English studies will be excluded due to the constraints of time, cost, and translator availability.
Studies relating to the development of interpersonal and communication skills in non-preceptorship nursing education	The focus of this review is to establish current educational practices for the development of interpersonal and communication skills in preceptorship education and training programmes. Therefore, studies that are not specifically part of a preceptorship education and training programme will not be included.

### Stage 4: Data charting

In this stage, a data extraction form will be created by the lead author (PH) ( Table 4) based on JBI (2020) data charting form, mapping it with the objectives and research question of the scoping review ( Peters *et al*., 2020) and piloted on two articles by all authors. Any changes to the chart will be documented and reported in the final scoping review for transparency in the reporting. 

**Table 4.** Sample data charting form.

Data chart heading	Description
Author	Name of author/s
Date	Date article sourced
Title of article	Title of the article or study
Publication year	The year that the article was published
Publication type	Journal, website, conference, etc.
Study details	Type of study, empirical or review, etc.
Study Aim	The aims of the study
Research Design	The framework of research methods and techniques chosen by the researcher/s.
Methodological Approaches	Approach taken to examine the topic
Data Analysis	Analysis of data
Keywords	What keywords were present
Study setting	Country/hospital/programme
Nursing field	General, Children’s, Mental Health, Intellectual Disability, Midwifery
Study population	The population studied with regard to demographics
Theoretical framework	What educational frameworks were implemented to offer a distinctive way to frame teaching practices of IP & C skills
Pedagogical methods	Pedagogical methods applied to teaching IP & C skills
Educational strategies	Whether they include both preceptors and students or preceptors alone
Reported challenges or limitations	What challenges were encountered
Findings	Noteworthy results of the study
Conclusion	Important aspects of the conclusion

### Stage 5: Collating, summarising and reporting the results

Each data charting form will be logged electronically using Microsoft Excel to capture relevant information for each study and will be available for all members of the research team via a shared drive. All authors will discuss the data before a descriptive analysis commences. As recommended by Peters *et al*. (2020), the analysis of data extracted should not involve any more than descriptive analysis to achieve the desired outcomes of a scoping review. Therefore, a narrative report will be produced, using a deductive thematic analysis approach summarising the extracted data concerning the objectives and scoping review question, for example, the pedagogy adopted for interpersonal and communication skills development and the impact of such training on trainee preceptors. Identification of areas in which a gap in the literature exists will also be reported. Quality appraisal of studies will not be conducted, as this review aims to explore the general scope of research conducted in the field of interpersonal and communication skills development in preceptorship education and training programmes and identify current pedagogical practices implemented to contribute a theoretical and empirical basis for the future development of preceptorship education and training programmes.

### Stage 6: Consultation and dissemination

Initial findings from the scoping review will be presented to several stakeholders. The primary author (PH) will disseminate the results of the review with local academic networks within the authors’ place of work (third level institution) and associated clinical settings. The author will specifically report the findings to Clinical Placement Coordinators (CPC), who typically develop and facilitate preceptorship education and training days in the clinical settings in Ireland. The primary author will also share the results at the Clinical Skills Network of Ireland in which he is a stakeholder to reach a national targeted audience. The authors will engage with these groups to share and discuss our findings and interpretations to capture their perspective on the evidence identified. The primary author also aims to deliver an oral or poster presentation at National and International conferences such as the International Nursing & Midwifery Research and Education Conference, scheduled for March 2022. Finally, the authors aim to publish the scoping review findings in a peer-reviewed journal for a wider communication of the results. All data generated and analysed during the scoping review will be included in the published scoping review article; including search results, list of included studies, data extraction spreadsheets and final results, to ensure transparency and reproducibility of the review.

### Study status

This study is at Stage 2 – a preliminary search of the literature has been conducted and the software packages Mendeley and Covidence have been trialled.

## Conclusions

This scoping review protocol has been designed in line with the latest literature and evidence ( Arksey & O’Malley’s, 2005; Peters *et al*., 2020; Tricco *et al*., 2018) to create and perform a systematic scoping review. The distinguishing features of a scoping review will permit the authors to answer the specified research question, applying a systematic and evidence-based approach to identify the current knowledge on educational practices for the development of interpersonal and communication skills as part of preceptorship education and training programmes. It will also enable the authors to identify gaps in our knowledge base in this field which could justify new research and also inform the design, conduct and reporting of future research. 

While this scoping review will not formally evaluate the quality of evidence available, it will provide a comprehensive overview of the available literature that will inform the researcher on current educational practices for the development of interpersonal and communication skills as part of preceptorship education and training programmes. This knowledge may identify the gaps in training that are contributing to interpersonal conflicts in preceptorship relationships that are widely reported throughout the literature. Only articles in English will be utilised; however, there will be no restrictions on the country of origin where the publications were produced, which should therefore provide a diverse range of opinions, experiences and cultural contexts. Following the open peer-review process and achieved approval, the authors will commence the systematic scoping review.

## Data availability

No data are associated with this article.

## References

[ref-52] Arksey H O'Malley L: Scoping studies: towards a methodological framework. *Int J Soc Res Methodol.* 2005;8(1):19–32. 10.1080/1364557032000119616

[ref-1] Arnold E Boggs K: Interpersonal Relationships. Professional Communication Skills for Nurses, 8th Edition, Elsevier, St Louis, Missouri, USA,2020. Reference Source

[ref-50] Billay D Myrick F: Preceptorship: An integrative review of the literature. *Nurse Educ Pract.* 2008;8(4):258–266. 10.1016/j.nepr.2007.09.005 17988946

[ref-62] Carlson E: Precepting and symbolic interactionism – a theoretical look at preceptorship during clinical practice. * J Adv Nurs.* 2013;69(2):457–64. 10.1111/j.1365-2648.2012.06047.x 22670850

[ref-2] Cho SH Mark BA Knafl G: Relationships Between Nurse Staffing and Patients' Experiences, and the Mediating Effects of Missed Nursing Care. *J Nurs Scholarsh.* 2017;49(3):347–355. 10.1111/jnu.12292 28388827

[ref-3] Deering CG Eichelberger L: Mirror, Mirror on the Wall: Using Online Discussion Groups to Improve Interpersonal Skills. *Comput Inform Nurs.* 2002;20(4):150–154. 10.1097/00024665-200207000-00010 12105403

[ref-4] Dinç L Gastmans C: Trust in nurse-patient relationships: A literature review. *Nurs Ethics.* 2013;20(5):501–516. 10.1177/0969733012468463 23426234

[ref-5] Dyess SM Sherman RO: The first year of practice: new graduate nurses' transition and learning needs. *J Contin Educ Nurs.* 2009;40(9):403–10. 10.3928/00220124-20090824-03 19754027

[ref-6] Gardiner I Sheen J: Graduate nurse experiences of support: A review. *Nurse Educ Today.* 2016;40:7–12. 10.1016/j.nedt.2016.01.016 27125143

[ref-7] Grant A Goodman B: Communication and Interpersonal Skills in Nursing. 4th Edition, Sage Publications Limited, London, UK,2019. Reference Source

[ref-8] Holst H Ozolins LL Brunt D: The experiences of supporting learning in pairs of nursing students in clinical practice. *Nurse Educ Pract.* 2017;26:6–11. 10.1016/j.nepr.2017.06.002 28646680

[ref-9] Hugo L Botma Y: Looking beneath the surface of a preceptor-training programme through a realist evaluation. *Eval Program Plann.* 2019;73:195–203. 10.1016/j.evalprogplan.2019.01.005 30685736

[ref-10] Irwin C Bliss J Poole K: Does Preceptorship improve confidence and competence in Newly Qualified Nurses: A systematic literature review. *Nurse Educ Today.* 2018;60:35–46. 10.1016/j.nedt.2017.09.011 28987897

[ref-11] Jackson VA Back AL: Teaching communication skills using role-play: an experience-based guide for educators. *J Palliat Med.* 2011;14(6):775–80. 10.1089/jpm.2010.0493 21651366 PMC3155105

[ref-12] JBI Collaboration Handbook: Joanna Briggs Institute, Adelaide, Australia.2020. Reference Source

[ref-13] Ke YT Kuo CC Hung CH: The effects of nursing preceptorship on new nurses’ competence, professional socialization, job satisfaction and retention: A systematic review. *J Adv Nurs.*Blackwell Publishing Ltd.2017;73(10):2296–2305. 10.1111/jan.13317 28398636

[ref-14] Kornhaber R Walsh K Duff J: Enhancing adult therapeutic interpersonal relationships in the acute health care setting: an integrative review. *J Multidiscip Healthc.* 2016;9:537–546. 10.2147/JMDH.S116957 27789958 PMC5072574

[ref-15] Lin ECL Chen SL Chao SY: Using standardized patient with immediate feedback and group discussion to teach interpersonal and communication skills to advanced practice nursing students. *Nurse Educ Today.*Elsevier Ltd,2013;33(6):677–683. 10.1016/j.nedt.2012.07.002 22841362

[ref-16] Lockwood C dos Santos KB Pap R: Practical Guidance for Knowledge Synthesis: Scoping Review Methods. *Asian Nurs Res (Korean Soc Nurs Sci).*Elsevier BV.2019;13(5):287–294. 10.1016/j.anr.2019.11.002 31756513

[ref-17] MacLean S Kelly M Geddes F: Use of simulated patients to develop communication skills in nursing education: An integrative review. *Nurse Educ Today.*Elsevier Ltd,2017;48:90–98. 10.1016/j.nedt.2016.09.018 27741440

[ref-18] Martínez-Linares JM Parra-Sáez C Tello-Liébana C: Should We Be Trained to Train? Nursing Students' and Newly Qualified Nurses' Perception on Good Lecturers and Good Clinical Preceptors. *Int J Environ Res Public Health.* 2019;16(24):4885. 10.3390/ijerph16244885 31817138 PMC6950704

[ref-19] McCloughen A Foster K: Nursing and pharmacy students' use of emotionally intelligent behaviours to manage challenging interpersonal situations with staff during clinical placement: A qualitative study. *J Clin Nurs.* 2018;27(13–14):2699–2709. 10.1111/jocn.13865 28426909

[ref-20] McConnell CR: Interpersonal skills. What they are, how to improve them, and how to apply them. *Health Care Manag (Frederick).* 2004;23(2):177–87. 10.1097/00126450-200404000-00012 15192999

[ref-21] McGowan J Straus S Moher D: Reporting scoping reviews-PRISMA ScR extension. *J Clin Epidemiol.* 2020;123:177–179. 10.1016/j.jclinepi.2020.03.016 32229248

[ref-22] McKenzie JE Brennan SE Ryan RE: Chapter 3: Defining the criteria for including studies and how they will be grouped for the synthesis. In: Higgins JPT, Thomas J, Chandler J, Cumpston M, Li T, Page MJ, Welch VA (editors). *Cochrane Handbook for Systematic Reviews of Interventions*version 6.1 (updated September 2020). Cochrane,2020. Reference Source

[ref-63] McSharry E: An exploration of clinical teaching and learning within a preceptorship model in an acute care hospital in the Republic of Ireland. EdD thesis The Open University2013. 10.21954/ou.ro.0000bfb9

[ref-23] McSharry E Lathlean J: Clinical teaching and learning within a preceptorship model in an acute care hospital in Ireland; a qualitative study. *Nurse Educ Today.* 2017;51:73–80. 10.1016/j.nedt.2017.01.007 28130976

[ref-24] Moss B: Communication skills in Nursing, Health and Social Care. 5th Edition, Sage Publications Limited, London, UK,2020. Reference Source

[ref-25] Muir J Ooms A Tapping J: Preceptors’ perceptions of a preceptorship programme for newly qualified nurses. *Nurse Educ Today.*Elsevier Ltd,2013;33(6):633–638. 10.1016/j.nedt.2013.02.001 23473751

[ref-26] Mukumbang FC Adejumo O: Patients’ experiences of being nursed by student nurses at a teaching hospital. *Curationis.* 2014;37(1):1230. 10.4102/curationis.v37i1.1230 25686403

[ref-28] NMC: Standards framework for nursing and midwifery education. Nursing & Midwifery Council, UK,2018. Reference Source

[ref-27] Nursing and Midwifery Board of Ireland: Nurse Registration Programmes Standards and Requirement. 4th edn. Nursing and Midwifery Board of Ireland, Dublin, Ireland,2016a. Reference Source

[ref-29] Nursing and Midwifery Board of Ireland: Quality Learning Environment. Nursing and Midwifery Board of Ireland, Dublin, Ireland.2016b. Reference Source

[ref-51] Omer TY Moola SH: The importance of the Preceptor-Preceptee Relationship in Creating Well Prepared Professionals: A Make or Break Experience. *Glob J Health Sci.* 2019;1(1). 10.5539/gjhs.v11n1p1

[ref-53] Page MJ Moher D: Evaluations of the uptake and impact of the Preferred Reporting Items for Systematic reviews and Meta-Analyses (PRISMA) Statement and extensions: a scoping review. *Syst Rev.* 2017;6(1):263. 10.1186/s13643-017-0663-8 29258593 PMC5738221

[ref-31] Pavord E Donnelly E: Communication and Interpersonal skills.2nd Edition, Lantern Publishing Ltd UK,2015. Reference Source

[ref-32] Pearson E McLafferty I: The use of simulation as a learning approach to non-technical skills awareness in final year student nurses. *Nurse Educ Pract.* 2011;11(6):399–405. 10.1016/j.nepr.2011.03.023 21497554

[ref-33] Perry J Watkins M Gilbert A: A systematic review of the evidence on service user involvement in interpersonal skills training of mental health students. *J Psychiatr Ment Health Nurs.* 2013;20(6):525–540. 10.1111/j.1365-2850.2012.01955.x 22845684

[ref-34] Peters MDJ Marnie C Tricco AC: Updated methodological guidance for the conduct of scoping reviews. *JBI Evid Synth.* 2020;18(10):2119–2126. 10.11124/JBIES-20-00167 33038124

[ref-35] Prip A Møller KA Nielsen DL: The Patient-Healthcare Professional Relationship and Communication in the Oncology Outpatient Setting: A Systematic Review. *Cancer Nurs.* 2018;41(5): E11–E22. 10.1097/NCC.0000000000000533 28753191 PMC6259679

[ref-36] Purdie A Sheward L Gifford E: Student nurse placements take a new direction. *Nurse Educ Pract.* 2008;8(5):315–320. 10.1016/j.nepr.2008.01.001 18291722

[ref-37] Reid-Searl K Quinney L Dwyer T: Puppets in an acute paediatric unit: Nurse’s experiences. *Collegian.* 2017;24(5):441–447. 10.1016/j.colegn.2016.09.005

[ref-38] Sloan G: Audio recording nurse-patient interactions. *Nurs Stand.* 2003;17(29):33–7. 10.7748/ns2003.04.17.29.33.c3367 12715577

[ref-39] Suikkala A Leino-Kilpi H: Nursing student-patient relationship: Experiences of students and patients. *Nurse Educ Today.* 2005;25(5):344–354. 10.1016/j.nedt.2005.03.001 15916836

[ref-40] Suikkala A Koskinen S Leino-Kilpi H: Patients’ involvement in nursing students’ clinical education: A scoping review. *Int J Nurs Stud.* 2018;84:40–51. 10.1016/j.ijnurstu.2018.04.010 29763831

[ref-41] Suikkala A Leino-Kilpi H Katajisto J: Nursing student-patient relationship and related factors-A self-assessment by nursing students. *J Clin Nurs.* 2020a;29(21–22):4030–4044. 10.1111/jocn.15426 32696592

[ref-42] Suikkala A Koskinen S Katajisto J: Congruence between nursing students’ and patients’ views of student-patient relationships. *Adv Health Sci Educ Theory Pract.* 2020b. 10.1007/s10459-020-09972-z 32436071 PMC7900057

[ref-43] Tricco AC Lillie E Zarin W: PRISMA Extension for Scoping Reviews (PRISMA-ScR): Checklist and Explanation. *Ann Intern Med.* 2018;169(7):467–473. 10.7326/M18-0850 30178033

[ref-44] Ward A McComb S: Precepting: A literature review. *J Prof Nurs.* 2017;33(5):314–325. 10.1016/j.profnurs.2017.07.007 28931478

[ref-45] Washington GT: The theory of interpersonal relations applied to the preceptor-new graduate relationship. *J Nurses Prof Dev.* 2013;29(1):24–9, quiz E1-2 10.1097/NND.0b013e31827d0a8a 23486153

[ref-46] Waugh A McNay L Devar B: Supporting the development of interpersonal skills in nursing, in an undergraduate mental health curriculum: Reaching the parts other strategies do not reach through action learning. *Nurse Educ Today.*Elsevier Ltd,2014;34(9):1232–1237. 10.1016/j.nedt.2013.10.002 25095983

[ref-47] Wright B: Interpersonal Skills: Skills for Caring. M&K Update Ltd Leeds, UK.2007; 1907830375,9781907830372. Reference Source

[ref-61] Vae KJU Engström M Mårtensson G: Nursing students' and preceptors' experience of assessment during clinical practice: A multilevel repeated-interview study of student–preceptor dyads. * Nurse Educ Pract.* 2018;30:13–19. 10.1016/j.nepr.2017.11.014 29475154

