# Supplementary material for: Interpersonal and communication skills development in nursing preceptorship education and training programmes: a scoping review protocol
Source: HRB Open Res. 2021 Mar 11;4:9. Originally published 2021 Jan 28. [Version 2] doi: 10.12688/hrbopenres.13201.2 (PMC7903201; doi:10.12688/hrbopenres.13201.2)
Supplement: Peer review history

# Peer review: Reviewer response for version 1

**DOI:** 10.21956/hrbopenres.14350.r28840

**Published:** 2021-02-24

**Authors:** Karen Poole

**Affiliations:** 1 Faculty of Health and Medical Sciences, School of Health Sciences, University Of Surrey, Guildford, UK

Thank you for inviting me to review this protocol. The authors make a compelling case for conducting a scoping review on the pedagogic practices used to develop communication and interpersonal skills in nurse preceptors. This protocol draws upon the most recent guidance for the conduct of scoping reviews, with a clear and well written account of the planned search strategies, data extraction and dissemination plans. 

 In terms of "context" scope, it may be helpful to clarify if you are including the educational preparation of preceptors for supporting Newly Qualified Nurses taking part in preceptorship programmes as well as undergraduate nursing programmes.

 I agree with Elisabeth Carlson (first reviewer) regarding the difficulty of applying the concept of the therapeutic relationship to the preceptor and nursing student. There are characteristics that are relevant, but I am not sure whether it is a faithful representation of this concept. You may wish to consider a minor revision to this paragraph? Preceptors have a critical role in shaping students' clinical experiences, but are also responsible for assessing their developmental progress both formatively and summatively (often in a placement of short duration). As such, there is a complex relationship between preceptors and nursing students and the use of effective communication and interpersonal skills (in both parties) is essential in negotiating learning opportunities and navigating safe honest formative feedback/feed-forward that enables students to optimise learning in practice placements through their programmes.

 Here are a couple of minor suggestions for inclusion in your plans for data extraction:

- capture of "nursing field" (learning disability, mental health, child, adult).
- capture of whether the educational strategies include both preceptors and students or preceptors alone.

This scoping review has the potential to make an important contribution in shaping how preceptors are prepared and support the future nursing workforce.

Is the study design appropriate for the research question?

Yes

Is the rationale for, and objectives of, the study clearly described?

Yes

Are sufficient details of the methods provided to allow replication by others?

Yes

Are the datasets clearly presented in a useable and accessible format?

Not applicable

Reviewer Expertise:

Education of Healthcare Professionals, Integrated Programmatic Assessment, Self-regulated Learning, Teaching Evidence-based Practice, Cancer Care.

I confirm that I have read this submission and believe that I have an appropriate level of expertise to confirm that it is of an acceptable scientific standard.

# Response

**Published:** 2021-03-03

**Authors:** Philip Hardie

The authors would like to thank you for your comments on our scoping review protocol and for your suggestions.

 We have responded to your comments below:

- A broader definition of preceptorship will be provided which will be more aligned to the diverse range of roles and responsibilities associated with a nursing preceptorship.
- We acknowledge the therapeutic relationship better describes the relationship between the nurse and patient and have amended the text accordingly.
- The “context” of the scope has been updated to include the educational preparation of preceptors for supporting Newly Qualified Nurses taking part in preceptorship programmes as well as undergraduate nursing programmes

# Peer review: Reviewer response for version 1

**DOI:** 10.21956/hrbopenres.14350.r28783

**Published:** 2021-02-23

**Authors:** Edel McSharry

**Affiliations:** 1 Department of Nursing, Health Science and Disability Studies, National University of Ireland Galway, Galway, Ireland; 2 St. Angela's College Lough Gill Sligo, Lough Gill, Ireland

The preceptor holds a dual role of practitioner and teacher. The preceptor must utilise complex teaching strategies to the foster the student’s ability to practise nursing competently and compassionately. One of the core competencies inherent in all nursing programmes is the student's ability to communicate effectively and develop positive professional interpersonal relationship with both patients and other health care professionals. It is essential that the preceptor trainee is proficient in these skills in order to be able to provide patient centered care and utilise the teaching techniques of role modelling, coaching and contextual questioning to facilitate the student’s learning. Students often find these skills a challenge to learn and preceptors often undervalue their own professional interpersonal communication skills ( Mallik et al 2009 ^1^, Mc Sharry 2013 ^2^).

 Preceptorship preparation varies in length and content and some studies have reported that preceptors do not feel adequately prepared for their teaching and assessment role. This scoping review focusing on interpersonal and communication skill development of preceptor trainees will contribute to existing literature that can inform the development of preparation programmes both nationally and internationally. It has the potential to contribute to pedological approaches that enhance both preceptor trainee skills and student’s interpersonal and communication skills. Any enhancement in these skills are can only positively contribute to the provision of quality person- centered care. The protocol is clearly written with well-defined aim and objectives, inclusion and exclusion criteria and appropriate search terms. It aligns wells to recent writings on methodological guidance for the conduct of scoping reviews.

 I have 3 suggestions that the authors may find useful in refining this protocol:

- Page 3 The definition of preceptorship offered is valuable and constitutes many of the attributes of the preceptorship model. Carlson (2013) definition develops on this definition and may be more appropriate. The preceptorship model in the Irish context further involves an assessment role which is not specified or encapsulated within these definitions as they are presented.
- Page 3 I would not entirely agree the preceptor- preceptee relationship is a therapeutic interpersonal relationship. This type of relationship is associated with a long term mentor- student relationship. The preceptor has to ensure the student gains the competencies required to pass the placement and therefore its focus is educational. The paper already sets out that the preceptor relationship is purposeful and short-term therefore I would suggest preceptorship involves a reciprocal student -preceptor relationship based on equality and mutual respect where the student’s confidence is fostered( Mc Sharry & Lathlean 2017 ^3^).
-  Page 3 and Page 4 there is a sentence that is repeated at the beginning of the paragraph at the bottom of page 3 and at the beginning of the paragraph on page 4. This repetition is not required and just requires some editing.

Is the study design appropriate for the research question?

Yes

Is the rationale for, and objectives of, the study clearly described?

Yes

Are sufficient details of the methods provided to allow replication by others?

Yes

Are the datasets clearly presented in a useable and accessible format?

Not applicable

Reviewer Expertise:

clinical education, digital learning, internationalisation

I confirm that I have read this submission and believe that I have an appropriate level of expertise to confirm that it is of an acceptable scientific standard.

## References

[1] : Mentorship in contemporary practice: the experiences of nursing students and practice mentors. *J Clin Nurs*.2008;17(14) : 10.1111/j.1365-2702.2007.02233.x 1834-42 10.1111/j.1365-2702.2007.02233.x 18578757

[2] : An exploration of clinical teaching and learning within a preceptorship model in an acute care hospital in the Republic of Ireland.2013; Reference source 10.1016/j.nedt.2017.01.00728130976

[3] : Clinical teaching and learning within a preceptorship model in an acute care hospital in Ireland; a qualitative study. *Nurse Educ Today*.2017;51: 10.1016/j.nedt.2017.01.007 73-80 10.1016/j.nedt.2017.01.007 28130976

# Response

**Published:** 2021-03-03

**Authors:** Philip Hardie

The authors would like to thank you for your comments on our scoping review protocol and for your suggestions.

 We have responded to your comments below:

- A broader definition of preceptorship will be provided which will be more aligned to the diverse range of roles and responsibilities associated with a nursing preceptorship.
- We acknowledge the therapeutic relationship better describes the relationship between the nurse and patient and have amended the text accordingly.

# Peer review: Reviewer response for version 1

**DOI:** 10.21956/hrbopenres.14350.r28841

**Published:** 2021-02-12

**Authors:** Elisabeth Carlson

**Affiliations:** 1 Department of Care Science, Faculty of Health and Society, Malmö University, Malmö, Sweden

The training and preparation of preceptors vary over the world. Some programs or rather initiatives are merely a couple of days or even hours long while others are full academic credit bearing courses at universities. This implies that studies on preceptor preparation is a subject always worthy of investigation. The protocol is well written, easy to follow and uses current methodological references. I applaud the authors that despite their educational context being Ireland, the protocol is written in such a way that it is easily transferable to an international context. I have three minor  comments or rather thoughts that might be useful.  

 Keywords: Alphabetical order

 Page 3 Definition (the quote): While this is very true and a frequently used definition, I would also recommend the more elaborated definition to be found in CARLSON E. (2013) Precepting and symbolic interactionism – a theoretical look

 at preceptorship during clinical practice. Journal of Advanced Nursing 69(2), 457– 464. doi: 10.1111/j.1365-2648.2012.06047.x ^1^

 Page 3: I am not quite sure if I agree that there is a therapeutic relationship between preceptor an nursing student. I would say there should be a strong educational and trustful professional relationship which in turn enables therapeutic interpersonal relationships with patients.

Is the study design appropriate for the research question?

Yes

Is the rationale for, and objectives of, the study clearly described?

Yes

Are sufficient details of the methods provided to allow replication by others?

Yes

Are the datasets clearly presented in a useable and accessible format?

Not applicable

Reviewer Expertise:

Higher Health Care Education, Learning theories, Preceptorship, Clinical Training, Methodology, Interprofessional Collaboration and learning, Educational models.

I confirm that I have read this submission and believe that I have an appropriate level of expertise to confirm that it is of an acceptable scientific standard.

## References

[1] : Precepting and symbolic interactionism--a theoretical look at preceptorship during clinical practice. *J Adv Nurs*.2013;69(2) : 10.1111/j.1365-2648.2012.06047.x 457-64 10.1111/j.1365-2648.2012.06047.x 22670850
